# Role of Neuropoietic Cytokines in Development and Progression of Diabetic Polyneuropathy: From Glucose Metabolism to Neurodegeneration

**DOI:** 10.1155/EDR.2003.303

**Published:** 2003

**Authors:** Dusanka S. Skundric, Robert P. Lisak

**Affiliations:** 1 Department of Neurology, Division of Neuroimmunology and Department of Immunology and Microbiology Wayne State University School of Medicine Detroit Medical Centre 421 E. Canfield, 3141 Elliman Building Detroit Michigan 48201 USA

## Abstract

Diabetic neuropathy develops as a result of hyperglycemia- induced local metabolic and microvascular changes in both type I and type II diabetes mellitus. Diabetic neuropathy shows slower impulse conduction, axonal degeneration, and impaired regeneration. Diabetic neuropathy affects peripheral, central, and visceral sensorimotor and motor nerves, causing improper locomotor and visceral organ dysfunctions. The pathogenesis of diabetic neuropathy is complex and involves multiple pathways. Lack of success in preventing neuropathy, even with successful treatment of hyperglycemia, suggests the presence of early mediators between hyperglycemia-induced metabolic and enzymatic changes and functional and structural properties of Schwann cells (SCs) and axons. It is feasible that once activated, such mediators can act independently of the initial metabolic stimulus to modulate SC-axonal communication. Neuropoietic cytokines, including interleukin-1 (IL-1), interleukin-6 (IL-6), leukemia inhibitory factor (LIF), ciliary neurotrophic factor (CNTF), tumor necrosis factor alpha (TNF-*α*), and transforming growth factor beta (TGF- *β*), exhibit pleiotrophic effects on homeostasis of glia and neurons in central, peripheral, and autonomic nervous system. These cytokines are produced locally by resident and infiltrating macrophages, lymphocytes, mast cells, SCs, fibroblasts, and sensory neurons. Metabolic changes induced by hyperglycemia lead to dysregulation of cytokine control. Moreover, their regulatory roles in nerve degeneration and regeneration may potentially be utilized for the prevention and/or therapy of diabetic neuropathy.


*Experimental Diab. Res.*, 4:303–312, 2003
Copyright © Taylor and Francis Inc.
ISSN: 1543-8600 print / 1543-8619 online
DOI: 10.1080/15438600390249736

Taylor & Francis
Taylor & Francis Group

### Role of Neuropoietic Cytokines in Development and Progression of Diabetic Polyneuropathy: From Glucose Metabolism to Neurodegeneration

**Dusanka S. Skundric and Robert P. Lisak**

*Department of Neurology, Division of Neuroimmunology, and Department of Immunology and Microbiology, Wayne State University School of Medicine, Detroit Medical Centre, Detroit, Michigan, USA*

**Diabetic neuropathy develops as a result of hyperglycemia-induced local metabolic and microvascular changes in both type I and type II diabetes mellitus. Diabetic neuropathy shows slower impulse conduction, axonal degeneration, and impaired regeneration. Diabetic neuropathy affects peripheral, central, and visceral sensorimotor and motor nerves, causing improper locomotor and visceral organ dysfunctions. The pathogenesis of diabetic neuropathy is complex and involves multiple pathways. Lack of success in preventing neuropathy, even with successful treatment of hyperglycemia, suggests the presence of early mediators between hyperglycemia-induced metabolic and enzymatic changes and functional and structural properties of Schwann cells (SCs) and axons. It is feasible that once activated, such mediators can act independently of the initial metabolic stimulus to modulate SC-axonal communication. Neuropoietic cytokines, including interleukin-1 (IL-1), interleukin-6 (IL-6), leukemia inhibitory factor (LIF), ciliary neurotrophic factor (CNTF), tumor necrosis factor alpha (TNF-$\alpha$), and transforming growth factor beta (TGF-$\beta$), exhibit pleiotrophic effects on homeostasis of glia and neurons in central, peripheral, and autonomic nervous system. These cytokines are produced locally by resident and infiltrating macrophages, lymphocytes, mast cells, SCs, fibroblasts, and sensory neurons. Metabolic changes induced by hyperglycemia lead to dysregulation of cytokine control. Moreover, their regulatory roles in nerve degeneration and regeneration may potentially be utilized for the prevention and/or therapy of diabetic neuropathy.**

**Keywords** Cytokines; Diabetic Neuropathy; Schwann Cell

### INTRODUCTION

Diabetic polyneuropathy is a chronic complication of both type I and type II diabetes mellitus. It affects peripheral, central, and visceral sensorimotor and motor nerves. Diabetic neuropathy develops as a result of hyperglycemia-induced local metabolic and microvascular changes. Chronic complications, including neuropathy, retinopathy, and nephropathy, are major causes of morbidity and mortality in patients with type I and type II diabetes (review by Sheetz and King, 2002; Williams et al., 2002).

In peripheral nerve, abnormalities of the $Na^+$ and $K^+$ transport along myelinated axons lead to impairment of the nerve function. Insufficient function of the peripheral nerve is manifested as slower conduction velocity, which develops early after the onset of diabetes. This early functional abnormality is followed by more pronounced changes in the morphology of Schwann cells (SCs), axons, and sensory neurons, which ultimately lead to axonal degeneration. Studies from experimental models that resemble the patterns of human pathology provide insights into the metabolic and molecular pathways underlying diabetic neuropathy (Sima et al., 2000, Sima, 2001; review by Eckersley, 2002). Homeostasis of myelinated peripheral nerve fibers is maintained through the SC-axonal contacts and communications. This includes, but is not limited to, production

Received 13 February 2003; accepted 11 June 2003.
Address correspondence to Dusanka S. Skundric, MD, PhD, Department of Neurology, Wayne State University School of Medicine, 421 E. Canfield, 3141 Elliman Building, Detroit, MI 48201, USA. E-mail: skundric@cmb.biosci.wayne.edu of neuropoietic cytokines and neurotrophic factors, expression of cell adhesion molecules (CAMs) and deposition of extracellular matrix (ECM) by SCs. In diabetic neuropathy, many aspects of SC-axonal communication are compromised. Disturbed local homeostasis leads to axonal degeneration accompanied by impaired ability to regenerate (Xu et al., 2002). Conventional therapy of diabetes and maintenance of euglycemic levels combined with auxiliary therapies to increase perfusion and/or prevent aberrant pathways of glucose metabolism have failed to halt and/or restore functional and structural changes of peripheral nerve, in both humans and experimental animals. This striking lack of success in preventing neuropathy, even with successful treatment of hyperglycemia, suggests the presence of early mediators between hyperglycemia-induced metabolic and enzymatic changes and functional and structural properties of SCs and axons. It is feasible that once activated, such mediators can act independently of the initial metabolic stimulus to modulate SC-axonal communication. Partial success of some therapies in abrogating signs of already developed diabetic neuropathy argues against the development of completely irreversible changes, and provides hope for the discovery of novel therapies targeting early factors involved in pathogenesis of diabetic neuropathy. It is clear that the pathogenesis of diabetic neuropathy is complex and involves multiple pathways. Therefore, early therapeutic intervention with combined therapies aimed at modulation and/or blocking of aberrant pathways might prove to be beneficial in the prevention and/or reversal of diabetic neuropathy. Such an approach is further emphasized by findings that combined blocking of several pathways, independently induced by high glucose, is needed for restoration of the metabolic abnormalities that underlie microvascular complications in diabetes. This therapeutic strategy has proved successful in the treatment of experimental diabetic retinopathy (Nishikawa, 2000; Hammes et al., 2003).

In this article we will briefly summarize major properties of a specific group of mediators called neuropoietic cytokines within the peripheral nervous system (PNS) and present evidence for their role in the regulation of homeostasis, glucose metabolism, degeneration, and regeneration of the peripheral nerve. Neuropoietic cytokines, including interleukin-1 (IL-1), interleukin-6 (IL-6), leukemia inhibitory factor (LIF), ciliary neurotrophic factor (CNTF), tumor necrosis factor alpha (TNF-$\alpha$) and transforming growth factor beta (TGF-$\beta$), are potent regulators of homeostatic properties of peripheral nerve. These cytokines are produced locally by resident and infiltrating macrophages, lymphocytes, mast cells, SCs, fibroblasts, and sensory neurons. Metabolic changes induced by hyperglycemia lead to dysregulation of cytokine control.

### METABOLIC CHANGES IN THE DIABETIC PERIPHERAL NERVE

Elevated blood glucose levels activate aberrant pathways of glucose metabolism, such as the polyol pathway. Activation of such metabolic pathways leads to accumulation of their specific end products, such as sorbitol and fructose, and secondarily perturbs phosphoinositide turnover and diacylglycerol (DAG), which in turn may directly or indirectly cause damage to glia and neurons. Furthermore, local accumulation of these end products induces changes in cell signaling in glia and neurons, perturbing various homeostatic functions regulated by those cell-signaling pathways (review by Sheetz and King, 2002).

The hallmarks of glucose metabolism through the polyol pathway are activation of the enzyme aldose reductase (AR) and accumulation of sorbitol. Depending on its intracellular concentration, sorbitol may have either toxic or hyperosmotic effects on cells. Increased intracellular osmotic pressure, together with perturbed balances and exchange of cations and anions, affects transcription of numerous genes, including genes of proinflammatory cytokines IL-1, IL-6, and TNF-$\alpha$. However, therapies aimed at inhibiting AR used for the treatment and/or prevention of diabetic neuropathy in humans have failed to achieve desired effects. Similar therapies, if started early in different animal models of diabetic neuropathy, have, however, shown promising results in ameliorating neuropathy (review by Sima, 2001; Simmons and Feldman, 2002).

Hyperglycemia leads to accelerated phosphoinositide turnover, activation of phospholipase D and changes of DAG levels (Sugimoto et al., 2000). Although hyperglycemia in peripheral nerve leads to overall impaired phosphoinositide turnover, DAG accumulation and total protein kinase C (PKC) expression (Eichberg, 2000), there is evidence to suggest that vascular PKC-$\beta$ isoforms are increased in diabetic nerve. Activated phospholipase D and DAG induce changes in the activation of PKC and expression of its isoforms in peripheral nerve. Changes in PKC activation and/or PKC isoform expression can modulate the activity of $Na^+$, $K^+$-ATPase. The $Na^+$,$K^+$-ATPase pump actively regulates intracellular concentrations of these two cations important for the function of cellular enzymes and transcription factors, such as activator protein-1 (AP-1) and nuclear factor kappa B (NF-$\kappa$B). Transcription factors AP-1 and NF-$\kappa$B are involved in the initiation of the transcription of numerous genes, including proinflammatory and neuropoietic cytokines (IL-1, IL-6, TNF-$\alpha$). In SCs, stimulation of PKC through extracellular signal regulated kinase (ERK) activation regulates the levels of LIF mRNA (Nagamoto-Combs et al., 1999). Neuropoietic cytokines have pleiotrophic effects on glia and neurons, either directly or indirectly through regulation of neurotrophic factors. The binding of cytokines to the gp130 receptor activates the signal transducer and activators of transcription 3 (STAT3), mitogen-activated protein kinase (MAPK), MAPK kinase (MEK), phosphatidylinositol 3-kinase (PI3K), and serine/threonine kinases called Akt (protein kinase B) signaling pathways. These signaling pathways play important roles in the regulation of cellular glucose metabolism and cell survival. STAT3 and PI3K/Akt signaling play major roles in mediating the survival response of neurons to cytokines (Alonzi et al., 2001). The same signaling pathways may be activated by insulin itself and synergistically so by C-peptide (Grunberger et al., 2001; Li et al., 2002).

Lack of neurotrophic support by nerve growth factor (NGF), neurotropin-3 (NT-3), brain-derived nerve factor (BDNF), insulin-like growth factor-I (IGF-I), and vascular endothelial growth factor (VEGF) is implicated in apoptosis of dorsal root ganglia sensory neurons and axonal degeneration in diabetes (Thrailkill, 2000; Carmeliet and Storkebaum, 2002; Nitta et al., 2002). Systemic therapies aimed to supplement local trophic factor deficiency have shown limited or no effects (Pradat et al., 2001; Wellmer et al., 2001) in the protection and/or repair of diabetic peripheral nerve. In vitro experiments in which SCs were cultured in the presence of elevated glucose concentration revealed alterations in arachidonic acid metabolism. Decreased content of glycerophospholipid arachidonoyl-containing molecular species (ACMS), decreased free cytosolic nicotinamide adenine dinucleotide ($NAD^+$/NADH), increased malondialdehyde, depleted glutathione levels, and reduced cytosolic superoxide dismutase activity suggest that high glucose level elicit oxidative stress in SCs cultures (Miinea et al., 2002; Vincent et al., 2002). Oxidative stress and inflammatory cytokines influence the bidirectional relationship between iron metabolic pathways and glucose metabolism, amplifying and potentiating hyperglycemia-induced events (Fernandez-Real et al., 2002). In peripheral nerve, cytokines regulate functions essential for SC-axonal homeostasis and nerve regeneration, such as production of ECM, expression of CAMs, and organization of the cytoskeleton (Chandross et al., 1996). Lack of CAM expression by SCs and/or dysregulation of ECM production and deposition by SCs lead to improper contact between SCs and axons (Stewart et al., 1997). If not corrected, these abnormalities may cause degenerative changes such as fiber loss and perinodal demyelination (Fu and Gordon, 1997). Regulation of $Na^+$ and $K^+$ voltage-gated channel expression and function by proinflammatory cytokines has been reported in central nervous system (CNS) (Vega et al., 2002). The existence of similar mechanisms in peripheral nerve and their relevance for impulse propagation remain to be investigated (Figure 1).

### NEUROPOIETIC CYTOKINES: ROLE IN HOMEOSTASIS, DEGENERATION, AND REGENERATION OF PERIPHERAL NERVE AND RELATION TO GLUCOSE METABOLISM

Neuropoietic cytokines from the IL-1 and IL-6 families, TNF-$\alpha$, and TGF-$\beta$ exhibit pleiotrophic effects on glia cells and neurons important for the homeostasis of the peripheral, central, and autonomic nervous systems (Armati and Pollard, 1996; Benveniste, 1998; Chalazonitis et al., 1998; Charon et al., 1998; Geissen et al., 1998; Horton et al., 1998; Lisak et al., 1997). In this section, we will focus on cytokines produced locally by SCs and/or sensory neurons in the PNS and their effects on regulation of homeostasis and degeneration and regeneration of peripheral nerve. The effects of cytokines on similar functions in the CNS and autonomic nerves have been studied to a lesser extent.

Cytokines produced in peripheral nerve originate from resident and infiltrating macrophages, lymphocytes, mast cells, SCs, fibroblasts, and probably neurons. Cytokines are involved in the pathogenesis of nerve damage as well as repair. For example, TNF-$\alpha$ injected into nerve induces inflammatory demyelination and wallerian degeneration, whereas IL-1 production promotes phagocytosis by scavenger macrophages and the synthesis of neurotrophic factors nerve growth factor (NGF) and LIF (Lindholm et al., 1987; Carlson et al., 1996). After experimental axotomy, other neuropoietic cytokines, including IL-6, LIF, and TGF-$\beta$1, are overexpressed in nerve and promote axonal growth until the contact with SCs is established. Proinflammatory cytokines are instrumental to the course of inflammatory demyelinating neuropathies. They increase vascular permeability of the blood-nerve barrier, which favors transmigration of leukocytes into nerve. They induce activation and proliferation of lymphocytes and macrophages and may have a direct myelinotoxic activity. In addition, downregulation of the immunosuppressive cytokine TGF-$\beta$1 may favor the nerve inflammatory reactions (reviewed by Creange et al., 1997).

Mechanisms leading to nerve degeneration and regeneration are essential in the pathogenesis of diabetic neuropathy. Compared to normal, the ability of diabetic peripheral nerve to regenerate is significantly diminished (Cameron and Cotter, 1997). In a comparative analysis, Pierson et al. (2003) found that regenerative potentials in type I are affected to a greater extent than in type II diabetic neuropathy. The regulatory roles of cytokines in nerve degeneration and regeneration may potentially be utilized for the prevention and/or therapy of diabetic neuropathy. Prior to attempting cytokine-based therapy, one needs to understand the effects of long-term hyperglycemia on the local cytokine network. Data are still lacking regarding

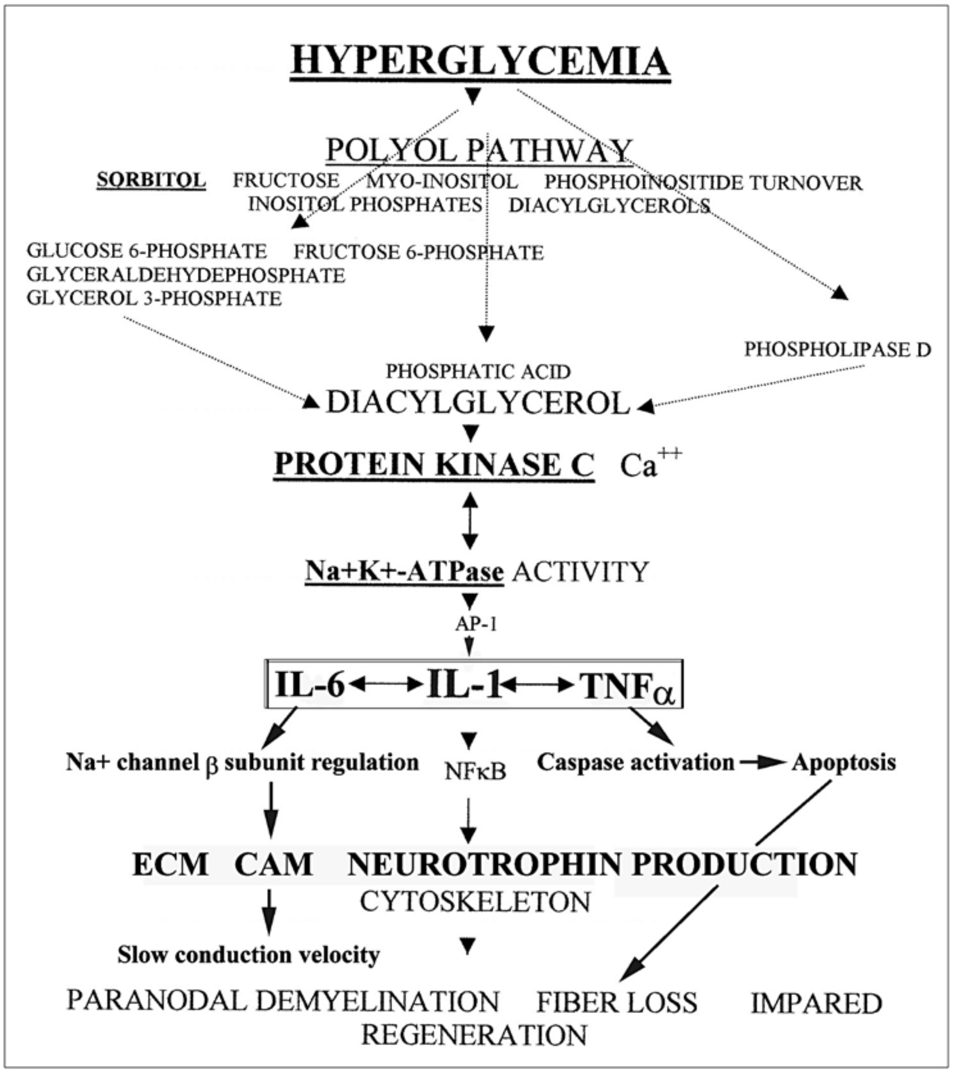

**FIGURE 1**

Schematic diagram of major aberrant metabolic pathways induced by hyperglycemia. The proposed role of proinflammatory cytokines in relation to metabolic end products, activated enzymes, transcription factors, neuropoietic cytokines, and molecules involved in Schwann cell–axonal communication.

the mechanisms involved in cytokine regulation by long-term hyperglycemia in peripheral nerve. Therefore, this review will focus on bringing together existing knowledge about major regulatory properties of neuropoietic cytokines in nerve degeneration and regeneration, and how these may be of relevance for diabetic neuropathy. Each cytokine family will be discussed separately because of many shared properties within family members. Some properties are shared between different cytokines and therefore cytokine redundancy plays an important role in the local cytokine network. Finally we will incorporate data from studies on cytokine regulation in diabetic neuropathy, which are still sparse but emerging. In different experimental models of diabetic neuropathy, only a handful of laboratories, including ours, have directly studied the regulation of cytokines and its role in the pathogenesis of diabetic neuropathy. Therefore, for the most part, we will summarize data from different models of nerve degeneration and regeneration, which are relevant to diabetic neuropathy, attempting to underscore the significance of further understanding of cytokine regulation in similar processes pertaining to diabetic neuropathy.

### Autocrine Regulation of IL-1 in Peripheral Nerve: Role in Homeostasis, Regeneration and the Pathogenesis of Diabetic Neuropathy

The SC is the myelin-producing glia of the PNS. It is responsible for myelination and ensheathment of myelinated peripheral nerve, fibers. SCs also provide trophic support for neuronal cells by regulation of neurotrophin, production in both normal and pathologic states. IL-1 is a proinflammatory and neuropoietic cytokine, locally produced by SCs, endoneurial and infiltrating macrophages and fibroblasts in peripheral nerve, and sensory neurons in dorsal root ganglia (Lisak et al., 1997). IL-1 regulates the production of other cytokines, neurotrophins, and pain mediators by SCs and sensory neurons (Shadiack et al., 1993). IL-1 induces production of NGF (Lindholm et al., 1987). Operating as autocrine and paracrine factors, locally produced proinflammatory cytokines, including IL-1, IL-6 and TNF-$\alpha$, interact through a cytokine-specific network in the PNS. Constitutive expression of IL-1$\alpha$, IL-1$\beta$, interleukin beta–converting enzyme (ICE, caspase-1), and type I IL-1 receptor (IL-1RI) has been detected in normal adult sciatic nerve. Proinflammatory cytokines are up-regulated by peripheral nerve injury, including contusion, transection, toxicity, autoimmune, and metabolic insults. Rapid up-regulation of cytokines following injury to peripheral nerve precedes macrophage infiltration and secretion of other products of inflammation. IL-1 is especially important for the regulation of macrophage chemoattraction by stimulation of macrophage chemoattractant protein-1 (MCP-1, CCL2) by SCs. SCs of denervated nerves attract macrophages by secretion of MCP-1 in a process regulated by IL-6 and LIF (Tofaris et al., 2002). LIF mRNA is virtually undetectable in uninjured sensory neurons but is induced by the inflammatory cytokine IL-1. Biological properties of IL-1 are mediated through IL-1RI, which has been suggested to stimulate a ceramide-dependent protein kinase pathway, much like TFN-$\alpha$ (Dinarello and Thompson, 1991) IL-1 regulation of LIF mRNA, through stimulation of the endosomal, acidic sphingomyelinase pathway, leads to ceramide activation of PKC zeta (Carlson and Hart, 1996). IL-1$\beta$ induction of LIF gene expression is at least partially transcriptional, but LIF mRNA increases to a greater extent than LIF transcription, suggesting a post-transcriptional regulation as well (Carlson et al., 1996).

IL-1$\beta$, IL-6, and LIF affect dorsal root ganglia neurons in terms of survival or neuritogenesis. Some of the effects are indirect, probably mediated by NGF (Edoff and Jerregard, 2002). SCs regulate IL-1 production in an autocrine manner. They produce both IL-1$\alpha$ and IL-1$\beta$ and a natural antagonist, IL-1 receptor antagonist (IL-1RA). SCs also express IL-1R I, a biologically active IL-1 receptor (Skundric et al., 1997).

The ability of SCs to autoregulate IL-1 is activated in local immune regulation during autoimmune demyelinating neuritis (Figure 2) (Skundric et al., 2001). We investigated IL-1 autoregulation in sciatic nerves of BB/W spontaneously diabetic rats throughout the course of diabetic neuropathy. Our results showed activation of IL-1 signaling pathway in SCs, prior to and during the early stages of diabetic neuropathy, thus suggesting a role in the initiation of abnormal SC-axonal interaction (Skundric et al., 2002a). Activation of ICE (caspase-1) corresponded to up-regulation of IL-1$\beta$ production and recruitment of IL-1 receptor associated kinase (IRAK) by SCs at the early stages of neuropathy. Moreover, activation of IL-1 signaling was occurring concomitantly with activation of I$\kappa$B and subsequent translocation of NF-$\kappa$B p65 in SCs (Skundric et al., 2002a). Activation of IL-1 signaling pathway may impact on the regulation of other proinflammatory cytokines in diabetic nerve. It may also be involved in the regulation of oxidative metabolism through regulation of inducible nitric oxide synthase (iNOS) and therefore reactive oxygen species (ROS). Oxidative stress has been recently proposed as a mechanism responsible for apoptosis induction in diabetic neuropathy (Vincent et al., 2002).

Taken together, IL-1 is a potent regulator of both homeostatic mechanisms relevant for peripheral nerve function and IL-1 signaling, involved in pathogenic mechanisms implicated in the development of diabetic neuropathy. Activation of IL-1 signaling in diabetic nerve may thus represent initial compensatory effects and, if sustained, may contribute to ongoing degeneration. Future experiments designed to examine functional and structural changes in diabetic nerve induced by local modulation of IL-1 signaling are needed to explore these potential effects. The IL-1 cytokine family and components of IL-1–specific signaling cascade have been thoroughly described and many transgenic mice models have been generated to facilitate the study of IL-1 pathways. Unfortunately, the utilization of transgenic mice is of limited use for experiments required to understand the role of IL-1 in diabetic neuropathy. Such experiments have been mainly hampered by the significant role of proinflammatory cytokines in the pathogenesis of type I diabetes. Therefore, strategies to modulate cytokine production and signaling locally need to be explored. Such approaches are feasible in the case of IL-1; the basis for experimental therapies may include local use of the natural antagonist IL-1RA, specific signaling blockers, and IL-1–specific traps.

### NEUROPOIETIC PROPERTIES OF IL-6, LIF, AND CNTF: REGULATION IN DIABETIC NERVE

IL-6, CNTF, LIF, and cardiotrophin-1 (CT-1) comprise a group of structurally related cytokines that promote the survival of subsets of neurons in the developing peripheral nervous system through activation of transcription factor NF-$\kappa$B (Middleton et al., 2000). Constitutive expression of IL-6, IL-6 receptors, and the signaling protein of the IL-6 receptor, gp130, suggests a role in the homeostasis of the peripheral nerve. IL-6 is induced in peripheral nerve following injury and it is produced by SCs (Bolin et al., 1995). The role of neuropoietic cytokines

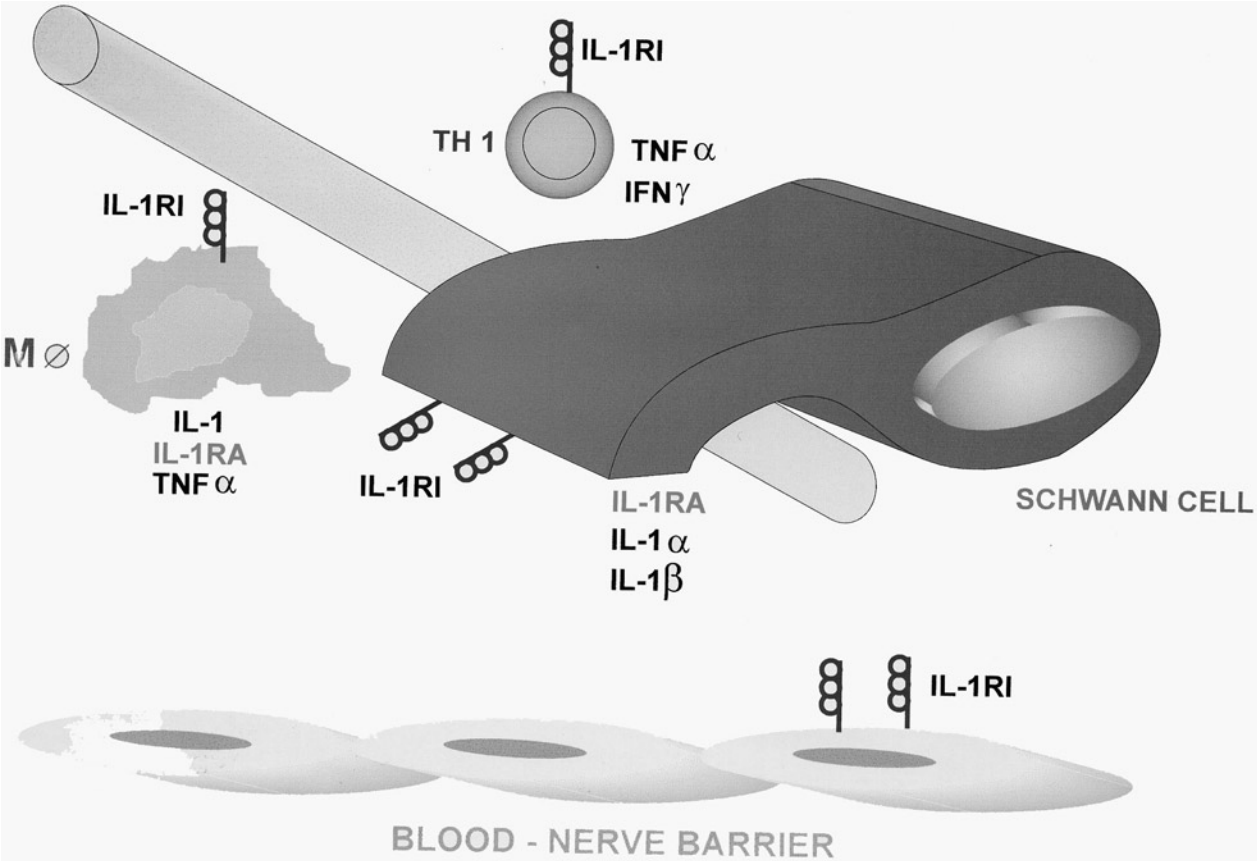

**FIGURE 2**

Schematic diagram presenting IL-1 autoregulation by Schwann cells, macrophages (Mo), and T lymphocytes (Th1) in the peripheral nerve during experimental autoimmune neuritis (EAN).

of the IL-6 family in the homeostasis and regeneration of the peripheral nerve can be modulated by neurotrophins. This regulation is bidirectional and influences the regenerative ability of the peripheral nerve. For example, NGF inhibits sympathetic neurons' response to LIF through regulation of injury-induced molecules, such as galanin. Galanin expression is triggered by two consequences of nerve transection: the induction of LIF and the reduction in the availability of the target-derived factor, NGF (Shadiack et al., 1998). Similarly, damage-induced neuronal endopeptidase (DINE/ECEL) expression is regulated by LIF induction and deprivation of NGF in rat sensory ganglia after nerve injury (Kato et al., 2002).

In human chronic inflammatory demyelinating polyneuropathy (CIDP), multiple neurotrophic growth factors and cytokines, NGF, glial cell line-derived neurotrophic factor (GDNF), LIF, and IL-6, are expressed together with their concomitant receptors in the nerve lesions and play an important role particularly in nerve repair (Yamamoto et al., 2002). The fact that the expression of neuropoietic cytokines and their receptors correspond to similarities in pathologic, events, rather than a specific disease, is clear from analyses of various human peripheral neuropathies (Ito et al., 2001). LIF appears to regulate IGF-I expression in the peripheral nerve in the basal state and early in the regeneration response in vivo (de Pablo et al., 2000). There is no direct evidence that LIF and CNTF are regulated by hyperglycemia. Future studies should reveal if mechanisms of LIF and CNTF regulation in diabetic neuropathy correspond to patterns observed in other degenerative and demyelinating neuropathies. Such data, combined with known mechanisms of neurotrophic factor regulation in diabetic neuropathy, may be useful in considering combined therapies aimed at ameliorating degeneration and improving regeneration of diabetic nerve.

Our results from gene array analysis of BB/W rats, which spontaneously develop type I diabetes accompanied with diabetic neuropathy, showed concomitant down-regulation of voltage-gated $Na^+$ channel $\beta$ subunits with IL-6 expression and signaling through STAT3 at early stages of diabetic neuropathy (Skundric et al., 2002b; Sima et al., 2003). Similar down-regulation of $Na^+$ channel $\beta$ subunits was observed in SCs cultured in hyperglycemic condition. Levels of $Na^+$ channel $\beta2$ and $\beta3$ subunits were recovered by addition of IL-6 to SCs cultures (Skundric et al., 2002c). These subunits play important roles as CAMs and in distribution and anchoring of voltage-gated $Na^+$ channels at the nodal axolemma (Isom, 2002; Sima et al., 2003). Lateralization of voltage-gated $Na^+$ channels from the node and their spreading to the juxtaparanodal and internodal regions have been observed in diabetic nerves of rats with type I diabetes (Cherian et al., 1996). Therefore, the potential of IL-6 to restore $\beta$-subunit expression may provide a basis for modulation of two processes essential for peripheral nerve integrity: one, related to voltage-gated $Na^+$ channel anchoring in the membrane and, second, CAM expression by SCs. IL-6 may play an important role in reestablishing CAM expression and binding to appropriate ECM ligands, which is profoundly perturbed in diabetic nerve (Merry et al., 1998) (see Figure 1). Proper contact between SCs through CAM expression with ECM is necessary for SC-axonal homeostatic communication. We also found that IL-6 exhibits the potential to regulate the expression of $Na^+$,$K^+$-ATPase in cultured SCs (Skundric, unpublished data). These findings underscore the significance of understanding the mechanisms of IL-6–specific regulation of voltage-gated $Na^+$ channels and $Na^+$,$K^+$-ATPase in peripheral nerve. Future experiments will be required to reveal if IL-6 modulates expression of only $\beta$ subunits, with CAM-like properties, or if it also affects the expression and function of $\alpha$ subunits, directly engaged in $Na^+$ and $K^+$ transport. A recent report from Sima et al. (2003) suggests lack of significant differences in expression levels of $\alpha$ subunits of voltage-gated $Na^+$ channels in diabetic neuropathy in rats.

### Role of TNF in Peripheral Nerve Degeneration, Apoptosis, and Glucose Metabolism

Experiments in mice with genetically distinct patterns of wallerian degeneration show that TNF-$\alpha$ and IL-1$\alpha$ initiate molecular and cellular events in rapid-wallerian degeneration (e.g., the production of additional cytokines and NGF). TNF-$\alpha$, IL-1$\alpha$, and IL-1$\beta$ may further regulate indirectly macrophage recruitment, myelin removal, regeneration, and neuropathic pain. In contrast to rapid-wallerian degeneration, production of TNF-$\alpha$, IL-1$\alpha$, and IL-1$\beta$ proteins is deficient in slow-wallerian degeneration, although their mRNAs are expressed. mRNA expression and protein production of TNF-$\alpha$, IL-1$\alpha$, and IL-1$\beta$ are differentially regulated during rapid-wallerian degeneration (normal) and slow-wallerian degeneration (delayed), suggesting that mRNA expression by itself is not an indication of the functional involvement of cytokines in wallerien degeneration (Shamash et al., 2002).

IL-1$\alpha$ and TNF-$\alpha$ markedly stimulate glucose utilization in primary cultures of mouse cortical astrocytes. PI3K is essential for the effect of both IL-1$\alpha$ and TNF-$\alpha$, whereas the action of IL-1$\alpha$ also requires activation of the MAP kinase pathway. The $Na^+$,$K^+$-ATPase inhibitor ouabain prevents the IL-1$\alpha$– and TNF-$\alpha$–stimulated 2-deoxyglucose (2DG) uptake. These data suggest that $Na^+$ pump activity is a common target for the long-term metabolic action of cytokines, promoted by the activation of distinct signaling pathways (Vega et al., 2002). In cultured myocytes, TNF-$\alpha$ decreased fatty-acid transport protein 1 (FATP-1) mRNA, insulin receptor substrate-1 (IRS-1)–associated PI3K activity, and glucose uptake, resulting in severe diet-induced insulin resistance (Maeda et al., 2002).

TNF-$\alpha$ is locally produced by SCs and has a role in peripheral nerve regeneration and regulation of apoptosis (Armati and Pollard, 1996). Our analysis of diabetic sciatic nerves in the spontaneously diabetic BB/W rat revealed up-regulation of TNF-$\alpha$ as well as initiator and executive caspases at early stages of diabetes. Activation of one of the major executive caspases, caspase-3, was observed. These findings suggest a role of TNF-$\alpha$ in regulation of apoptosis in diabetic neuropathy (Skundric et al., 2003). Mitochondrial dysfunction and apoptosis play important roles in the pathogenesis of diabetic neuropathy (Srinivasan et al., 2000). Revealing the mechanisms of TNF-$\alpha$–specific modulations of degeneration, regeneration, and apoptosis in diabetic peripheral nerve will significantly foster the development of appropriate therapies aimed to control these processes locally. TNF-$\alpha$ is a good candidate to be considered as a possible therapeutic target because of the availability of TNF-$\alpha$ family proteins, soluble receptors, and blockers of some signaling components. TNF-$\alpha$–related therapies have already been used in treatment of other neurodegenerative and inflammatory processes.

### Role of TGF-$\beta$ in the Cytokine-Specific Network in Peripheral Nerve: Implication for Diabetic Neuropathy

The TGF-$\beta$ family of cytokines is involved in multiple pathways regulating SC and neuronal development, SC proliferation, production of neurotrophic factors, ECM deposition, and expression of CAMs.

Treatment with TGF-$\beta$1 leads to induction of LIF mRNA in cultured SCs. This effect is mediated through PKC. The effect of TGF-$\beta$1 on LIF induction is abrogated by experimental conditions favoring SC-neuronal contact and myelination. This indicates that following lesions of peripheral nerve, SCs could respond to TGF-$\beta$1 by producing LIF, which in turn has neuroprotective effect and promotes regeneration. Once SC-axonal contact is established, effect of TGF-$\beta$ on LIF production subsides (Matsuoka et al., 1997). In contrast to induction of LIF, TGF-$\beta$ has negative regulatory effect on the production of NT-3 mRNA at the site of peripheral nerve injury (Cai et al., 1999).

TGF-$\beta$ plays a role in SC communication with ECM by regulating alpha1beta1 integrin expression (Stewart et al., 1997). Binding of alpha1beta1 integrin, expressed by SC, to ECM ligands laminin/collagen, plays an essential role in SC development and differentiation. In regard to sensory neuronal development, endogenous TGF-$\beta$ plays a significant role in regulation of ontogenetic neuron death. Experiments in developing chick embryo showed proapoptotic effects of TGF-$\beta$ signaling on ontogenetic death of sensory and motor neurons. These experiments suggest a similar role of TGF-$\beta$ in neuronal death in a situation of target deprivation (Krieglstein et al., 2000). TGF-$\beta$1 and TNF-$\alpha$ induce SC death by apoptosis (Skoff et al., 1998). TGF-$\beta$ also exhibits effect on SC differentiation (Lisak et al., 2001). Similar role for TGF-$\beta$, in control of developmentally regulated apoptosis of the SC lineage, has been described. This role is executed via TGF-$\beta$–specific phosphorylation of the early response gene c-Jun in nondifferentiated SCs, whereas more differentiated SCs are protected from this mechanism (Parkinson et al., 2001). In relation to SC plasticity, which is critical for peripheral nerve regeneration, TGF-$\beta$ has beneficial effect by supporting the proliferating SC phenotype involved in axonal regeneration. It is important that supportive effects of TGF-$\beta$ on axonal regeneration overcome the negative effects of long-term SC denervation (Sulaiman and Gordon, 2002). Specific up-regulation of TGF-$\beta$, its receptors (type I and type II), and intracellular second messengers, Smad 2/3 and 4, was observed in sensory neurons and satellite cells of dorsal root ganglia following injury (Stark et al., 2001). Future experiments designed to reveal the molecular mechanisms of TGF-$\beta$ regulation in diabetic nerve are needed in order to explore the potential therapeutic utilization of TGF-$\beta$ in the prevention and treatment of diabetic neuropathy.

### CONCLUSION AND FUTURE DIRECTIONS

Diabetic polyneuropathy is a chronic debilitating complication of both type I and type II diabetes mellitus. It develops as a result of hyperglycemia-induced local metabolic and microvascular changes. Neuropathy affects peripheral, central, and visceral sensorimotor and motor nerves and causes impairment of locomotor system and visceral organs. Neuropathy, along with other chronic complications, is the leading cause of morbidity and mortality of diabetes patients.

The pathogenesis of diabetic neuropathy is complex and involves multiple pathways. Therefore, early therapeutic intervention with combined therapies aimed at modulation and/or blocking of aberrant pathways might prove to be beneficial in the prevention and/or reversal of diabetic neuropathy.

Neuropoietic cytokines from the IL-1 and IL-6 families, TNF-$\alpha$, and TGF-$\beta$ exhibit pleiotrophic effects on glia cells and neurons important for the homeostasis of the peripheral, central, and autonomic nervous systems. Cytokines regulate degeneration and regeneration of peripheral nerve, processes essential in the pathogenesis of diabetic neuropathy. The contribution of cytokines to homeostasis and repair is mediated through numerous functions, including regulation of neurotrophic factors, ECM, CAMs, and SC proliferation and differentiation.

The regulatory roles of cytokines in nerve degeneration and regeneration may potentially be utilized for the prevention and/or therapy of diabetic neuropathy. Prior to attempting cytokine-based therapy, one needs to understand the effects of long-term hyperglycemia on the local cytokine network. Neuropoietic cytokines and components of their specific signaling cascade have been thoroughly described and many transgenic mice models have been generated to facilitate the study of cytokine-specific pathways. Unfortunately, the utilization of transgenic mice is of limited use for experiments required to understand their role in diabetic neuropathy. Such experiments have been hampered mainly by the significant role of proinflammatory cytokines in the pathogenesis of type I diabetes. Therefore, strategies to modulate cytokine production and signaling locally need to be explored. Such approaches are feasible in the case of IL-1, IL-6, and TNF-$\alpha$. The basis for experimental therapies may include local use of the natural antagonists, soluble receptors, blocking antibodies, specific signaling blockers, and specific cytokine traps.

### REFERENCES

Alonzi, T., Middleton, G., Wyatt, S., Buchman, V., Betz, U. A., Muller, W., Musiani, P., Poli, V., and Davies, A. M. (2001) Role of STAT3 and PI 3-kinase/Akt in mediating the survival actions of cytokines on sensory neurons. *Mol. Cell Neurosci.,* **18,** 270–282.

Armati, P. J., and Pollard, J. D. (1996) Immunology of the Schwann cell. *Baillieres Clin Neurol.,* **5,** 47–64.

Benveniste, E. N. (1998) Cytokine actions in the central nervous system. *Cytokine Growth Factor Rev.,* **9,** 259–275.

Bolin, L. M., Verity, A. N., Silver, J. E., Shooter, E. M., and Abrams, J. S. (1995) Interleukin-6 production by Schwann cells and induction in sciatic nerve injury. *J. Neurochem.,* **64,** 850–858.

Cai, F., Campana, W. M., Tomlinson, D. R., and Fernyhough, P. (1999) Transforming growth factor-beta 1 and glial growth factor beta 2 reduce neurotrophin-3 mRNA expression in cultured Schwann cells via a camp-dependent pathway. *Brain Res. Mol. Brain Res.,* **71,** 256–264.

Cameron, N. E., and Cotter, M. A. (1997) Metabolic and vascular factors in the pathogenesis of diabetic neuropathy. *Diabetes,* **46 (Suppl.),** S31–S37.

Carlson, C. D., Bai, Y., Jonakait, G. M., and Hart, R. P. (1996) Interleukin-1 beta increases leukemia inhibitory factor mRNA levels through transient stimulation of transcription rate. *Glia,* **18,** 141–151.

Carlson, C. D., and Hart, R. P. (1996) Activation of acidic sphingomyelinase and protein kinase C zeta is required for IL-1 induction of LIF mRNA in a Schwann cell line. *Glia,* **18,** 49–58.

Carmeliet, P., and Storkebaum, E. (2002) Vascular and neuronal effects of VEGF in the nervous system: Implications for neurological disorders. *Semin. Cell Dev. Biol.,* **13,** 39–53.

Chalazonitis, A., Rothman, T. P., Chen, J., Vinson, E. N., MacLennan, A. J., and Gershon, M. D. (1998) Promotion of the development of enteric neurons and glia by neuropoietic cytokines: Interactions with neurotrophin-3. *Dev. Biol.,* **198,** 343–365.

Chandross, K., Spray, D., Cohen, R. I., Kumar, N. M., Kremer, M., Dermietzel, R., and Kessler, J. A. (1996) TNF-a inhibits Schwann cell proliferation, connexin46 expression, and gap junctional communication. *Mol. Cell Neurosci.,* **7,** 479–500.

Charon, I., Zuin-Kornmann, G., Bataille, S., and Schorderet, M. (1998) Protective effect of neurotrophic factors, neuropoietic cytokines and dibutyryl cyclic AMP on hydrogen peroxide-induced cytotoxicity on PC12 cells: A possible link with the state of differentiation. *Neurochem. Int.,* **33,** 503–511.

Cherian, P. V., Kamijo, M., Angelides, K. J., and Sima, A. A. F. (1996) Nodal $Na^+$ channel displacement is associated with nerve-conduction slowing in the chronically diabetic BB/Wrat: Prevention by aldose reductase inhibition. *J. Diabetes Complications,* **10,** 192–200.

Creange, A., Barlovatz-Meimon, G., and Gherardi, R. K. (1997) Cytokines and peripheral nerve disorders. *Eur. Cytokine Netw.,* **8,** 145–151.

Dinarello, C. A., and Thompson, R. C. (1991) Blocking IL-1 interleukin 1 receptor antagonist in vivo and in vitro. *Immunol. Today,* **12,** 404–410.

Eckersley, L. (2002) Role of the Schwann cell in diabetic neuropathy. *Int. Rev. Neurobiol.,* **50,** 293–321.

Edoff, K., and Jerregard, H. (2002) Effects of IL-1beta, IL-6 or LIF on rat sensory neurons co-cultured with fibroblast-like cells. *J. Neurosci. Res.,* **67,** 255–263.

Eichberg, J. (2002) Protein kinase C changes in diabetes: Is the concept relevant to neuropathy? *Int. Rev. Neurobiol.,* **50,** 61–82.

Fernandez-Real, J. M., Lopez-Bermejo, A., and Ricart, W. (2002) Cross-talk between iron metabolism and diabetes. *Diabetes,* **51,** 2348–2354.

Fu, S. Y., and Gordon, T. (1997) The cellular and molecular basis of peripheral nerve regeneration. *Mol. Neurobiol.,* **14,** 67–116.

Geissen, M., Heller, S., Pennica, D., Ernsberger, U., and Rohrer, H. (1998) The specification of sympathetic neurotransmitter phenotype depends on gp130 cytokine receptor signaling. *Development,* **125,** 4791–4801.

Grunberger, G., Xiaolong, Q., Li, Z. G., Mathews, S. T., Sbriessa, D., Shisheva, A., and Sima, A. A. F. (2001) Molecular basis for the insulimamimetic effects of C-peptides. *Diabetologia,* **44,** 1247–1257.

Hammes, H. P., Edelstein, D., Taguchi, T., Matsumura, T., Ju, Q., Lin, J., Bierhaus, A., Nawroth, P., Hannak, D., Neumaier, M., Bergfeld, R., Giardino, I., and Brownlee, M. (2003) Benfotiamine blocks three major pathways of hyperglycemic damage and prevents experimental diabetic retinopathy. *Nat. Med.,* **9,** 294–299.

Horton, A. R., Barlett, P. F., Pennica, D., and Davies, A. M. (1998) Cytokines promote the survival of mouse cranial sensory neurones at different developmental stages. *Eur. J. Neurosci.,* **10,** 673–679.

Isom, L. (2002) The role of sodium channels in cell adhesion. *Front. Biosci.,* **7,** 12–33.

Ito, Y., Yamamoto, M., Mitsuma, N., Li, M., Hattori, N., and Sobue, G. (2001) Expression of mRNAs for ciliary neurotrophic factor (CNTF), leukemia inhibitory factor (LIF), interleukin-6 (IL-6), and their receptors (CNTFR alpha, LIFR beta, IL-6R alpha, and gp130) in human peripheral neuropathies. *Neurochem. Res.,* **26,** 51–58.

Kato, R., Kiryu-Seo, S., and Kiyama, H. (2002) Damage-induced neuronal endopeptidase (DINE/ECEL) expression is regulated by leukemia inhibitory factor and deprivation of nerve growth factor in rat sensory ganglia after nerve injury. *J. Neurosci.,* **22,** 9410–9418.

Krieglstein, K., Richter, S., Farkas, L., Schuster, N., Dunker, N., Oppenheim, R.W., and Unsicker, K. (2000) Reduction of endogenous transforming growth factors beta prevents ontogenetic neuron death. *Nat. Neurosci.,* **3,** 1085–1090.

Li, Z.-G., Zhang, W., and Sima, A. A. F. (2002) C-peptide prevents hippocampal apoptosis in type I diabetes. *Int. J. Exp. Diabetes Res.,* **3,** 241–246.

Lindholm, D., Heumann, R., Meyer, M., and Thoenen, H. (1987) Interleukin-1 regulates synthesis of nerve growth factor in non- neuronal cells of rat sciatic nerve. *Nature,* **330,** 658.

Lisak, R. P., Bealmear, B., Benjamin, J. A., and Skoff, A. M. (2001) Interferon-gamma, tumor necrosis factor-alpha and transforming growth factor-beta inhibit cyclic AMP-induced Schwann Cell differentiation. *Glia,* **36,** 354–363.

Lisak, R. P., Skundric, S., Bealmear, B. and Ragheb, S. (1997) The role of cytokines in Schwann cell damage, protection and repair. *J. Infect. Dis.,* **176 (Suppl. 2),** S173–S179.

Maeda, N., Shimomura, I., Kishida, K., Nishizawa, H., Matsuda, M., Nagaretani, H., Furuyama, N., Kondo, H., Takahashi, M., Arita, Y., Komuro, R., Ouchi, N., Kihara, S., Tochino, Y., Okutomi, K., Horie, M., Takeda, S., Aoyama, T., Funahashi, T., and Matsuzawa, Y. (2002) Diet-induced insulin resistance in mice lacking adiponectin/ACRP30. *Nat. Med.,* **8,** 731–737.

Matsuoka, I., Nakane, A., and Kurihara, K. (1997) Induction of LIF-mRNA by TGF-beta 1 in Schwann cells. *Brain Res.,* **776,** 170–180.

Merry, A. L., Yamamoto, K., and Sima, A. A. F. (1998) Imbalances in N-CAM, SAM, and polysialic acid may underlie the paranodal ion channel barrier defect in diabetic neuropathy. *J. Diabetes Complications,* **40,** 153–160.

Middleton, G., Hamanoue, M., Enokido, Y., Wyatt, S., Pennica, D., Jaffray, E., Hay, R. T., and Davies, A. M. (2000) Cytokine-induced nuclear factor kappa B activation promotes the survival of developing neurons. *J. Cell Biol.,* **148,** 325–332.

Miinea, C., Kuruvilla, R., Merrikh, H., and Eichberg, J. (2002) Altered arachidonic acid biosynthesis and antioxidant protection mechanisms in Schwann cells grown in elevated glucose. *J. Neurochem.,* **81,** 1253–1262.

Nagamoto-Combs, K., Vaccariello, S. A., and Zigmond, R. E. (1999) The levels of leukemia inhibitory factor mRNA in a Schwann cell line are regulated by multiple second messenger pathways. *J. Neurochem.,* **72,** 1871–1881.

Nishikawa, T., Edelstein, D., Du, X. L., Yamagishi, S., Matsumura, T., Kaneda, Y., Yorek, M. A., Beebe, D., Oates, P. J., Hammes, H. P., Giardino, I., and Brownlee, M. (2000) Normalizing mitochondrial superoxide production blocks three pathways of hyperglycaemic damage. *Nature,* **404,** 787–790.

Nitta, A., Murai, R., Suzuki, N., Ito, H., Nomoto, H., Katoh, G., Furukawa, Y., and Furukawa, S. (2002) Diabetic neuropathies in brain are induced by deficiency of BDNF. *Neurotoxicol. Teratol.,* **24,** 695–701.

de Pablo, F., Banner, L. R., and Patterson, P. H. (2000) IGF-I expression is decreased in LIF-deficient mice after peripheral nerve injury. *Neuroreport,* **11,** 1365–1368.

Parkinson, D. B., Dong, Z., Bunting, H., Whitfield, J., Meier, C., Marie, H., Mirsky, R., and Jessen, K. R. (2001) Transforming growth factor beta (TGFbeta) mediates Schwann cell death in vitro and in vivo: Examination of c-Jun activation, interactions with survival signals, and the relationship of TGFbeta-mediated death to Schwann cell survival. *J. Neurosi.,* **21,** 8572–8585.

Pierson, C. R., Zhang, W., Murakawa, Y., and Sima, A. A. (2002) Early gene responses of trophic factors in nerve regeneration differ in experimental type 1 and type 2 diabetic polyneuropathies. *J. Neuropathol. Exp. Neurol.,* **61,** 857–871.

Pradat, P. F., Kennel, P., Naimi-Sadaoui, S., Finiels, F., Orsini, C., Revah, F., Delaere, P., and Mallet, J. (2001) Continuous delivery of neurotrophin 3 by gene therapy has a neuroprotective effect in experimental models of diabetic and acrylamide neuropathies. *Hum. Gene Ther.,* **12,** 2237–2249.

Shadiack, A. M., Hart, R. P., Carlson, C. D., and Jonakait, G. M. (1993) Interleukin-1 induces substance P in sympathetic ganglia through the induction of leukemia inhibitory factor (LIF). *J. Neurosci.,* **13,** 2601.

Shadiack, A. M., Vaccariello, S. A., Sun, Y., and Zigmond, R. E. (1998) Nerve growth factor inhibits sympathetic neurons' response to an injury cytokine. *Proc. Natl. Acad. Sci. U. S. A.,* **95,** 7727–7730.

Shamash, S., Reichert, F., and Rotshenker, S. (2002) The cytokine network of Wallerian degeneration: Tumor necrosis factor-alpha, interleukin-1alpha, and interleukin-1beta. *J. Neurosci.,* **22,** 3052–3060.

Sheetz, M. J., and King, G. L. (2002) Molecular understanding of hyperglycemia's adverse effects for diabetic complications. *JAMA,* **288,** 2579–2588.

Sima, A. A. F. (2001) Diabetic neuropathy; polero genetic backgrounds, current and future therapies. *Expert Rev. Neurotherapeut,* **1,** 225–238.

Sima, A. A. F., Pierson, C. R., and Zhang, W. (2003) C-peptide prevents the molecular abnormalities of the paranode in type I diabetic polyneuropathy. In: *Proceedings of the 18th International Diabetes Federation Congress*.

Sima, A. A. F., Zhang, W., Xu, G., Sugimoto, K., Guberski, D., and Yorek, M. A. (2000) A comparison of diabetic polyneuropathy in type II diabetic BBZDR/Wor rats and in type I diabetic BB/Wor rats. *Diabetologia,* **43,** 786–793.

Simmons, Z., and Feldman, E. L. (2002) Update on diabetic neuropathy. *Curr. Opin. Neurol.,* **15,** 595–603.

Skoff, A. M., Lisak, R. P., Bealmear, B., and Benjamins, J. A. (1998) TNF alpha and TGF-beta act synergistically to kill Schwann cells. *J. Neurosci. Res.,* **53,** 747–756.

Skundric, D. S., Bealmear, B., and Lisak, R. P. (1997) Induced upregulation of IL-1, IL-1RA and IL-1R type I gene expression by Schwann cells. *J. Neuroimmunol.,* **74,** 9–18.

Skundric, D. S., Dai, R., James, J., and Lisak, R. P. (2002a) Activation of IL-1 signaling pathway in Schwann cells during diabetic neuropathy. *Ann. N. Y. Acad. Sci.,* **951,** 393–398.

Skundric, D. S., Dai, R., James, J., Sima, A. A. F., and Lisak, R. P. (2002b) Hyperglycemia induced activation of Stat3 signaling pathway in Schwann cells (SCs). *Diabetes,* **Suppl. 2,** A523 (Abstract).

Skundric, D. S., Dai, R. J., Mataverde, P., Lam, J. S., Kahn, D. E., and Hart, R. P. (2002c) Role of IL-6 in modulation of $Na^+$ and $K^+$ transport at early stages of diabetic neuropathy. *Diabetes Metab. Res. Rev.,* **18 (Suppl. 4),** S23 (Abstract).

Skundric, D. S., Dai, R., and Mataverde, P. (2003) Regulation of TNF-$\alpha$ and caspase-3 in diabetic neuropathy. In: *Apoptosis 2003, From Signalling Pathways to Therapeutic Tools, Proceedings*, Edited by Diederich, M., pp. 347 (Abstract). Luxembourg, Fondation de Recherche Cancer et Sang.

Skundric, D. S., Lisak, R. P., Rouhi, M., Kieseiner, B. C., Jung, S., and Hartung, H. P. (2001) Schwann cell specific regulation of IL-1 and IL-1Ra during EAN: Possible relevance for immune regulation at paranodal regions. *J. Neuroimmunol.,* **116,** 74–82.

Srinivasan, S., Stevens, M., and Wiley, J. W. (2000) Diabetic peripheral neuropathy: Evidence for apoptosis and associated mitochondrial dysfunction. *Diabetes,* **49,** 1932–1938.

Stark, B., Carlstedt, T., and Risling, M. (2001) Distribution of TGF-beta, the TGF-beta type I receptor and the R-II receptor in peripheral nerves and mechanoreceptors; observations on changes after traumatic injury. *Brain Res.,* **913,** 47–56.

Stewart, H. J., Turner, D., Jassen, K. R., and Mirsky, R. (1997) Expression and regulation of alpha1beta1 integrin in Schwann cells. *J. Neurobiol.,* **33,** 914–928.

Sugimoto, K., Murakawa, Y., and Sima, A. A. F. (2000) Diabetic neuropathy—a continuing enigma. *Diabetes Metab. Res. Rev.,* **16,** 408–433.

Sulaiman, O. A., and Gordon, T. (2002) Transforming growth factor-beta and forskolin attenuate the adverse effects of long-term Schwann cell denervation on peripheral nerve regeneration in vivo. *Glia,* **37,** 206–218.

Thrailkill, K. M. (2000) Insulin-like growth factor-I in diabetes mellitus: Its physiology, metabolic effects, and potential clinical utility. *Diabetes Technol. Ther.,* **2,** 69–80.

Tofaris, G. K., Patterson, P. H., Jessen, K. R., and Mirsky, R. (2002) Denervated Schwann cells attract macrophages by secretion of leukemia inhibitory factor (LIF) and monocyte chemoattractant protein-1 in a process regulated by interleukin-6 and LIF. *J. Neurosci.,* **22,** 6696–6703.

Vega, C., Pellerin, L., Dantzer, R., and Magistretti, P. J. (2002) Long-term modulation of glucose utilization by IL-1alpha and TNF-alpha in astrocytes: $Na^+$ pump activity as a potential target via distinct signaling mechanisms. *Glia,* **39,** 10–18.

Vincent, A. M., Brownlee, M., and Russell, J. W. (2002) Oxidative stress and programmed cell death in diabetic neuropathy. *Ann. N. Y. Acad. Sci.,* **959,** 368–383.

Wellmer, A., Misra, V. P., Sharief, M. K., Kopelman, P. G., and Anand, P. (2001) A double-blind placebo-controlled clinical trial of recombinant human brain-derived neurotrophic factor (rhBDNF) in diabetic polyneuropathy. *J. Periph. Nerv. Syst.,* **6,** 204–210.

Williams, R., Van Gaal, L., and Lucioni, C. (2002) Assessing the impact of complications on the costs of type II diabetes. *Diabetologia,* **45,** S13–S17.

Yamamoto, M., Ito, Y., Mitsuma, N., Li, M., Hattori, N., and Sobue, G. (2002) Parallel expression of neurotrophic factors and their receptors in chronic inflammatory demyelinating polyneuropathy. *Muscle Nerve,* **25,** 601–604.

Xu, G., Pierson, C. R., Murakawa, Y., and Sima, A. A. F. (2002) Altered tubulin and neurofilament expression and impaired axonal growth in diabetic nerve regeneration. *J. Neuropathol. Exp. Neurol.,* **61,** 164–175.

